# Targeting Oxidative Stress Injury after Ischemic Stroke in Conscious Rats: Limited Benefits with Apocynin Highlight the Need to Incorporate Long Term Recovery

**DOI:** 10.1155/2013/648061

**Published:** 2013-01-14

**Authors:** Robert M. Weston, Bin Lin, Gregory J. Dusting, Carli L. Roulston

**Affiliations:** ^1^Stroke Injury and Repair Team, O'Brien Institute, St Vincent's Hospital Melbourne, Fitzroy, Victoria, Australia; ^2^Cytoprotection Pharmacology Program, Centre for Eye Research, The Royal Eye and Ear Hospital Victoria, Melbourne, Victoria, Australia; ^3^Department of Ophthalmology, Faculty of Medicine, The University of Melbourne, Victoria, Australia; ^4^Department of Surgery, Faculty of Medicine, The University of Melbourne, Victoria, Australia

## Abstract

NADPH oxidase is a major source of superoxide anion following stroke and reperfusion. This study evaluated the effects of apocynin, a known antioxidant and inhibitor of Nox2 NADPH, on neuronal injury and cell-specific responses to stroke induced in the conscious rat. Apocynin treatment (50 mg/kg i.p.) commencing 1 hour prior to stroke and 24 and 48 hours after stroke significantly reduced infarct volume in the cortex by *~* 60%, but had no effect on striatal damage or neurological deficits. *In situ* detection of reactive oxygen species (ROS) using dihydroethidium fluorescence revealed that increased ROS detected in OX-42 positive cells following ischemia was reduced in apocynin-treated rats by *~* 51%, but surprisingly increased in surrounding NeuN positive cells of the same rats by *~* 27%, in comparison to the contralateral hemisphere. Reduced ROS from activated microglia/macrophages treated with apocynin was associated with reduced Nox2 immunoreactivity without change to the number of cells. These findings confirm the protective effects of apocynin and indicate a novel mechanism via reduced Nox2 expression. We also reveal compensatory changes in neuronal ROS generation as a result of Nox2 inhibition and highlight the need to assess long term individual cell responses to inhibitors of oxidative stress.

## 1. Introduction

Oxidative stress contributes to brain reperfusion injury following stroke [1]. Well-established sources of reactive oxygen species (ROS) generation in the brain following injury include intracellular organelles (especially mitochondria), invading neutrophils, activated microglia/macrophages [2], and cerebral blood vessels [3]. Recently, we have also shown that neurons themselves generate large amounts of superoxide following transient stroke, an effect that contributes to the progression of injury over time [4]. 

Several drugs that target oxidative stress have been developed as potential therapies for ischemic stroke. Spin trap free radical inhibitors and antioxidants, including Ebselen (a glutathione peroxidase mimetic); NXY 059 (a nitrone-based free radical trapping agent); edaravone (a free radical scavenger) can reduce infarct volume in rodent reperfusion models of ischemic stroke, supporting the contribution of reactive oxygen species to ischemic damage following reperfusion [5]. These antioxidants, however, target reactive oxygen species only after they are formed and do not address the specific process by which these toxic molecules are generated. This is important for reactive oxygen species can rapidly cause damage before they are inactivated. Novel therapeutic strategies that target the source of reactive oxygen species generation may offer better neuroprotection. 

NADPH oxidase (Nox) is the major enzyme source of reactive oxygen species in the vascular system [6, 7], inflammatory cells [2], cerebral blood vessels [3, 8] and neurons [4, 9]. The NADPH oxidase complex generates the highly reactive free radical, superoxide, via its Nox2 catalytic subunit [6, 10], and is the active NADPH oxidase in inflammatory cells in humans and animals. There are 2 major isoforms of Nox2, with Nox1 and Nox4 differentially expressed depending on tissue type, and these play different roles in regulating reactive oxygen species production [7]. In the acute phase of stroke damage (1–7 days) following ischemia with reperfusion, we have previously demonstrated the Nox2 catalytic component to be the predominant Nox subtype that is upregulated and associated with phagocytic microglial cells, indicating this NADPH oxidase to be a potential target for therapeutic intervention [4]. 

Apocynin (4-hydroxy-3-methoxy-acetophenone) is a reported inhibitor of Nox1- and Nox2-dependent superoxide generation in peripheral inflammatory cells [11] and has been shown to reduce damage in animal models of global and focal cerebral ischemia [12–14]. Individual cell responses to stroke injury following apocynin treatment are yet to be fully explored. In the present study, we examine the effect of apocynin on cell-specific changes in NADPH oxidase and ROS generation in the brain following transient stroke in conscious rats and make the surprising finding that in addition to attenuating ROS generation in inflammatory cells, Nox2 protein expression is also down-regulated, whilst neuronal ROS is conversely increased.

## 2. Materials and Methods

### 2.1. Surgical Preparation

All animal experiments were performed in accordance with the Prevention of Cruelty to Animals Act 1986, under the guidelines of the National Health and Medical Research Council for the Care and Use of Animals for Experimental Purposes in Australia. The protocol was approved by the St Vincent's Hospital Animal ethics committee (AEC 31/06). All surgery was performed under general anesthesia, and all efforts were made to minimize suffering which included access to paracetomol (2 mg/kg in drinking water) for 24 hours prior to and following surgery, as well as extensive monitoring of each rat throughout the duration of the study. 

20 adult male Hooded Wistar rats weighing 300 g to 350 g (Laboratory Animal Services, The University of Adelaide, Australia) were included in this study. Rats were group-housed (4 rats to a cage), until endothelin-1-induced middle cerebral artery constriction, whereupon they were housed in separate cages under diurnal lighting with ambient temperature maintained between 20 and 22°C. Rats were given free access to food and water. Rats were anesthetized with pentobarbitone sodium (60 mg/kg dose i.p.) and a 23-gauge cannula was stereotaxically inserted into the piriform cortex 2 mm dorsal to the middle cerebral artery (0.2 mm anterior, −5.2 mm lateral, and −5.9 mm ventral) as previously described [4, 15, 16]. Following surgery, rats were allowed to recover for 5 days prior to stroke induction.

### 2.2. Stroke Induction

Focal cerebral ischemia was induced in the conscious rat by constriction of the right middle cerebral artery with perivascular administration of endothelin-1 (American Peptide Company; 60 pmol in 3 *μ*L saline over 10 min) via a 30-gauge injector needle that protruded 2 mm beyond the end of the previously implanted guide cannula [4, 15, 16]. During stroke induction, we observed counterclockwise circling, clenching, and dragging of the contralateral forepaw, validating the correct placement of the endothlin-1 cannula. Stroke severity was scored 0 to 5 according to our previously established protocol [16] with 5 being the most severe. Rats exhibiting a score of less than 3 were excluded from further study (*n* = 6 in total) as we have previously shown limited damage after stroke with scores below 3 [16]. Rectal temperature was measured using a thermistor probe, immediately prior to stroke and at 30 minute intervals after stroke for 5 hours. Each rat was weighed before stroke and daily after stroke.

### 2.3. Drug Treatment

Apocynin (Sigma, Sydney, Australia) (50 mg/kg given i.p.) or vehicle (10% DMSO and 90% sterile saline) was delivered 1 hour prior to stroke induction and then again at 24 and 48 hours after reperfusion (total of 3 × 50 mg/kg). The dosing regimen for apocynin was based on previous work by Tang and colleagues [12] in a rat model of transient cerebral ischemia. Pre-treatment of rats with apocynin or vehicle did not affect behavioural signs indicative of stroke induction and rats were assigned a stroke severity score during stroke to ensure that within each treatment group stroke ratings were equally matched and thus stroke severity evenly represented across all groups as in previous studies [16].

### 2.4. Assessment of Functional Outcome

Neurological abnormalities were evaluated with the use of a neurological deficit score that is based on detection of abnormal posture and hemiplegia described by Yamamoto and colleagues [17]. Briefly, the presence of thorax twisting and absence of contralateral forepaw extension when suspended by the tail was scored at 1 each. Hemiplegia was evaluated by placing the rats on a raised platform and where the contralateral forepaw and/or contralateral hind limb were observed to slip off the edge, a score of 1 was assigned for each. Thus, when scores were summed, the maximum neurological deficit score was 4 with a score of 0 considered normal. These functional assessment studies were conducted prior to any procedures (presurgery), immediately prior to endothelin-1 induced stroke (preischemia), and every day after stroke for the remainder of the study. The function of each rat was compared with prestroke scores, such that each rat acted as its own control. All rats were coded so that the investigator was blinded to the treatment condition.

### 2.5. Quantification of Ischemic Damage

Rats were decapitated 72 hours after stroke, forebrains removed, frozen over liquid nitrogen, and stored at −80°C prior to processing. Coronal forebrain sections (16 *μ*m) were serially cut in a cryostat (Leica Microsystems, Wetzlar, Germany) at eight predetermined planes throughout the brain (−3.2 to 6.8 mm relative to Bregma). The extent of brain injury following stroke was determined in triplicate unstained slide mounted sections using a micro computer imaging device (MCID M4 image analyzer, Imaging Research Inc., St. Catharines, ON, Canada) for ballistic light image analysis shown to permit rapid and reliable measurements of cellular damage according to the methods of Callaway and colleagues [18]. Total infarct volume was calculated by integrating the cross-sectional area of damage at each stereotaxic level with the distance between each of the levels. The influence of oedema was also corrected according to the methods of Callaway and colleagues [18].

### 2.6. *In Situ* Detection of Superoxide Using Dihydroethidium Fluorescence

The oxidative fluorescent indicator dihydroethidium (2 *μ*M; Molecular Probes, OR, USA) was used to evaluate production of ROS in slide mounted sections adjacent to those utilized above for identification of ischemic damage, as previously described and reported by others [4, 19]. Briefly, slides were incubated with (2 micromolar) dihydroethidium (0.1% DMSO in phosphate buffered normal saline (PBS) 0.1 M) in a humidified chamber and protected from light for 30 min at 37°C. In separate studies sections were also incubated with in the presence and absence of the mitochondrial inhibitor, rotenone (50 *μ*M), for 30 min at 37°C prior to co-incubation with dihydroethidium for 30 min at 37°C. All resulting sections were examined with a fluorescence microscope (Zeiss Axioskop2) equipped with a 590 nm long pass filter set for detection of red fluorescence (dihydroethidium). 

### 2.7. Quantification of Dihydroethidium Fluorescence

Multiple images were acquired from the ischemic core of the striatum and cortical infarct and from the corresponding regions in the contralateral hemisphere (i.e., the mirror image regions). Images were captured with a computer-controlled digital camera (Zeiss AxioCam MRc5 connected to AxioVision software) using the same fluorescence settings for all rats. The intensity of red fluorescence was calculated by tracing around individual cells and quantitatively analysed in ImageJ software [4]. The ratio of the ipsilateral to the contralateral intensity was calculated to assess the relative production of ROS in the stroke affected brain.

### 2.8. Fluorescence Immunohistochemistry with Dihydroethidium Double Labelling

In order to identify cell-specific ROS generation frozen sections adjacent to those used in dihydroethidium studies were preincubated for 1 hour in 10% normal Donkey serum prior to overnight incubation with neuronal mouse monoclonal anti NeuN (1 : 400; Millipore, North Ryde, Australia) or mouse monoclonal antibody MRC-II OX-42 for microglia (1 : 100; Serotec, Bicester, UK). Tissue sections were then washed in phosphate buffered saline (0.1 M, pH 7.4, 3 × 10 min) and transferred for incubation with the secondary antibody, Alexa Fluorophore 488 nm donkey anti-mouse IgG at 1 : 500 (Invitrogen, Mount Waverley, Australia) in phosphate buffered saline containing 2% normal goat serum and 0.3% Triton X-100 for a further 90 min at room temperature. Sections were then double labelled with dihydroethidium as described above for colocalization of superoxide and immunofluorescence. Sections were cover slipped using fluorescent mounting medium (Dako), and both red and green fluorescence emitted from dihydroethidium and each primary antibody, respectively, were detected using a fluorescent microscope equipped with both a 590 mm and 515–565 mm filter set (Zeiss, Axioskop2). Analogous experiments were also conducted with mouse IgG control serum in place of the primary antibody.

### 2.9. Nox2 Immunohistochemistry

For detection of Nox2 protein expression sections corresponding to those used above were treated separately with mouse monoclonal anti-gp91Phox (1 : 500; BD Biosciences, San Jose, CA, USA) as previously described [4] and analyzed using a standard HRP-based immunoassay using 3-amino-9-ethylcarbazole (AEC) mouse-to-mouse detection kit (Millipore). The color reactant was visualized with a Zeiss Axioskop4 microscope using bright field settings.

### 2.10. OX-42 (MRC-II) Immunohistochemistry

For morphological assessment of microglia reactivity, MRC-II OX-42 immunohistochemistry was visualized using cobalt-nickel-enhanced diaminobenzidine according to the protocol described previously [20]. Briefly, slide mounted sections were fixed in 4% paraformaldehyde for 30 min. All sections were subject to a serum-free protein block (Dako) prior to overnight incubation with the mouse monoclonal IgG OX-42 (MRC-II) primary antibody (1 : 1000; Serotec) at 4°C. Tissue sections were incubated with sheep anti-mouse biotinylated secondary antibody (1 : 500, Vector Laboratories) and subsequently streptavidin-conjugated horseradish peroxidase (1 : 500, Vector Laboratories) for 90 min each at room temperature. OX-42 positive cells were visualized following 20 min incubation with cobalt-nickel-enhanced diaminobenzidine (1 : 10; Pierce, Australia). The resulting sections were examined with a Zeiss Axioskop4 microscope using bright field settings.

### 2.11. Statistical Analyses

Neurological outcome data was analysed by nonparametric Kruskal-Wallis test. Physiological data and infarct volume was analyzed by two-way ANOVA for comparison between apocynin- and vehicle-treated groups, or for comparison between ipsilateral and contralateral sides following stroke. For analysis of dihydroethidium fluorescence and OX-42 counts within groups following stroke, a one-way repeated measures ANOVA (treatment x h after stroke) was performed. Individual comparisons were made using Tukey's test for all analyses where ANOVA yielded a significant result. We have previously shown that *n* = 6 are required for 80% power where a reduction in infarct by 30% is considered significant with a SD of approximately 15% for each group [16]. All data are given as mean ± SEM. A two-sided value of *P* < 0.05 was considered to be statistically significant. Statistical analyses and all graphical representations were generated using Graph Pad Prism 5.01 software.

## 3. Results

### 3.1. Effect of Apocynin on Physiological Variables

Rats did not lose weight as a result of surgery or stroke as in previous studies [16]. Rectal temperature prior to stroke was within normal physiological limits. Following stroke, induction rectal temperatures increased at 30 min in both treatment groups (*P* < 0.05; ANOVA with repeated measures for minutes after stroke), but returned to normal limits after this time (data not shown). This temperature increase at 30 min was less than 1°C for all groups and was, therefore, unlikely to induce nonischemic-related damage.

### 3.2. Effect of Apocynin on Functional Outcomes

**Figure 1:**
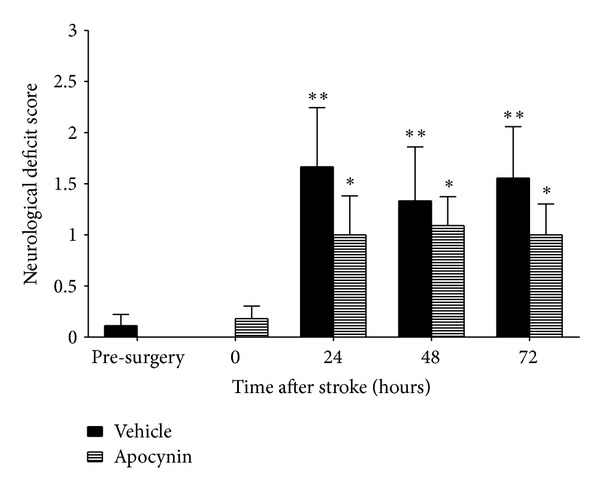
Effects of apocynin and vehicle treatments on functional outcome using the neurological deficit score. Data are presented as mean ± SEM (*n* = 11 apocynin treated; *n* = 9 vehicle treated). Each rat acted as its own control, with results after stroke compared to prestroke scores and between treatment groups at the same time. *P < 0.05; **P < 0.01, relative to presurgery and 0 h scores (ANOVA).

Neurological deficit tests were conducted in both treatment groups as a measure of functional outcome following stroke-induced brain injury. Endothelin-1-induced stroke resulted in significant neurological deficits 24 to 72 hours after stroke when compared to prestroke scores in all groups (*P* < 0.01; Figure 1). No significant difference existed between vehicle- or apocynin-treated rats at any time. 

### 3.3. Histopathological Analysis

**Figure 2:**
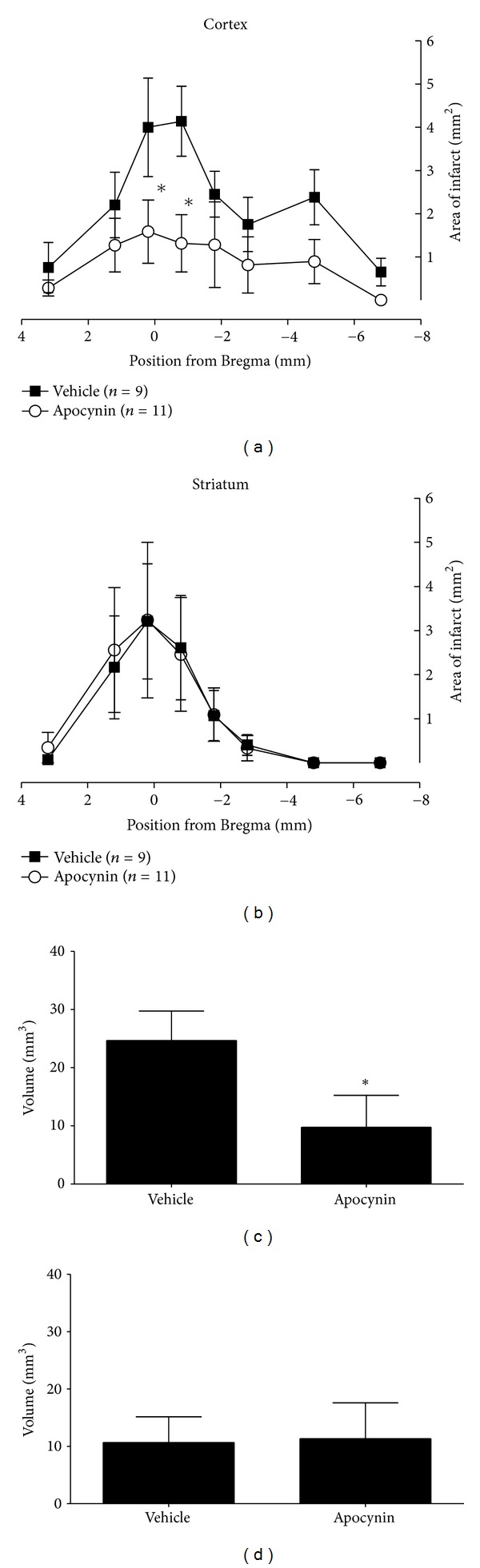
Effect of apocynin treatment on infarct area in cortex (a) and striatum (b) and total infarct volume in the cortex (c) and striatum (d) following stroke. Both infarct area and total volume were significantly reduced in the cortex following apocynin treatment, with no effect on the striatum. Data are presented as mean ± SEM (*n* = 11 apocynin treated; *n* = 9 vehicle treated). *P < 0.05 versus vehicle control rats (ANOVA).

Histological analysis 72 hours after stroke in vehicle-treated rats revealed damage in the parietal, insular, and frontal cortex, as well as in the dorsolateral striatum that extended through the striatum, typical to that reported previously [16] and to that reported with the use of other stroke models [21]. Treatment with apocynin significantly reduced infarct area through the cortex at more than one stereotaxic level when compared to vehicle treated control rats (*P* < 0.05 two-way ANOVA) (Figure 2(a)). Infarct volume in the apocynin treated rats was reduced by approximately 60% in the cortex, when compared to vehicle treatment (*P* < 0.01; ANOVA) (Figure 2(c)). Treatment with apocynin had no effect on damage to the striatum when compared to vehicle-treated rats.

### 3.4. *In Situ* Detection of Superoxide Using Dihydroethidium Fluorescence

The superoxide-sensitive indicator dihydroethidium was used to localize and quantitate the effect of apocynin on ROS generation from individual cells following stroke as in previous studies [4]. Regions of interest were identified using MCID generated images used to quantitate damage. Total dihydroethidium fluorescence was calculated by tracing around individual cells. The average dihydroethidium signal from all cells traced within the infarct was calculated and compared to the average of cells from the corresponding contralateral region (mirror image), which was expressed as 100% control.

**Figure 3:**
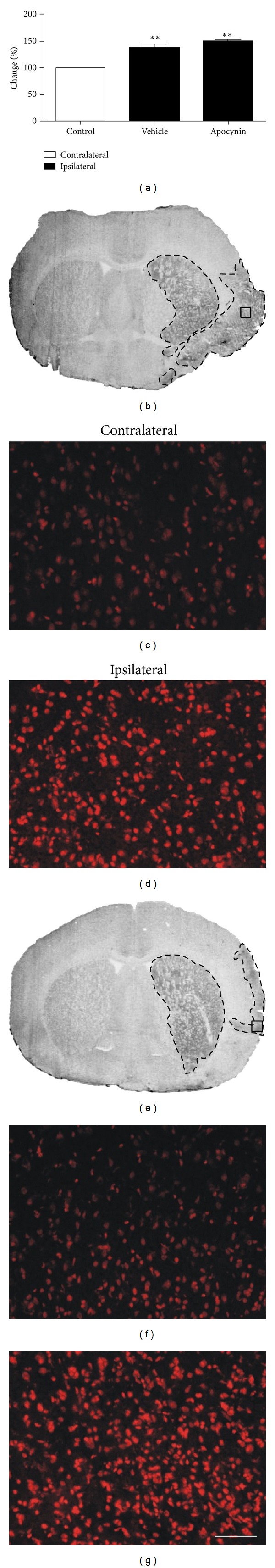
Quantification of superoxide production from all cells in the core infarct region detected *in situ* 3 days after stroke in the ipsilateral cortex (stroke affected; solid bars) compared with the contralateral cortex (nonaffected; open bar) following ischemic stroke in vehicle control and apocynin-treated rats (a). Core infarct regions of the cortex were identified using MCID images generated from unstained forebrain sections adjacent to those analyzed for superoxide, in vehicle control rats (b) and apocynin-treated rats (e). Representative fluorescence micrographs of sections incubated with superoxide-sensitive dihydroethidium in vehicle control ((c), (d)) and apocynin- ((f), (g)) treated rats from the ipsilateral cortex ((d), (g)) and the contralateral mirror image ((c), (f)). Relative fluorescence was quantified by tracing around individual cells and analysed using ImageJ software. Data are presented as mean ± SEM (*n* = 11 apocynin treated; *n* = 9 vehicle treated). **P < 0.01 versus contralateral control (ANOVA). Scale bar = 100 **μ**m.

Total dihydroethidium fluorescence within the ipsilateral cortex (Figures 3(d) and 3(g)) was significantly increased 3 days after stroke in vehicle (Figures 3(a) and 3(d)) and apocynin-treated rats (Figures 3(a) and 3(g)) when compared with the corresponding contralateral region (*P* < 0.01; Figures 3(a), 3(c), and 3(f), resp.). There was no difference in total fluorescence observed between treatment groups when all cells traced within each group were analysed together. Similarly, increased dihydroethidium fluorescence was also detected in the ipsilateral striatum from both vehicle- and apocynin-treated rats (data not shown).

### 3.5. Cell-Specific Localization of Superoxide Using Fluorescence Immunohistochemistry

**Figure 4:**
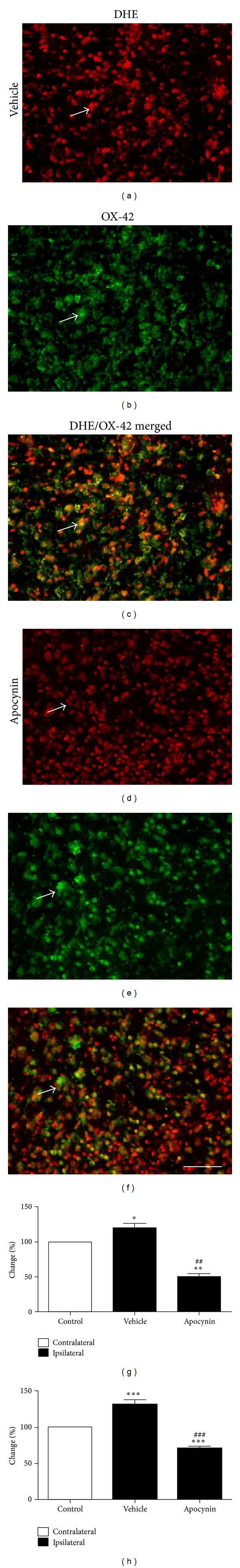
Immunohistochemical localization of activated microglia/macrophages (OX42) and concurrent superoxide generation (dihydroethidium) in stroke-affected brain following vehicle control ((a)–(c)) and apocynin treatments ((d)–(f)). Fluorescence micrographs of the ipsilateral cortex with superoxide sensitive dihydroethidium ((a), (d)), the microglia marker OX42 ((b), (e)) and merged images ((c, (f)). Arrows point to a typical activated microglia cell in the ischemic core from both treatment groups. Quantification of microglial specific superoxide release in the core infarct region detected *in situ* 3 days after stroke in the isilateral cortex (stroke affected; solid bars) compared with the contralateral cortex (nonaffected; open bar) in vehicle control and apocynin treated rats (g). Quantification of microglial specific superoxide release in the core infarct region in the ipsilateral striatum compared with the contralateral striatum in vehicle control and apocynin-treated rats (h). Relative fluorescence was quantified by tracing around cells double labelled with OX-42 and analysed using ImageJ software. Data are presented as mean ± SEM (*n* = 11 apocynin treated; *n* = 9 vehicle treated). *P < 0.05, **P < 0.01, and ***P < 0.001 versus contralateral mirror image; ^##^ *P* < 0.01; ^###^ *P* < 0.0001 versus vehicle-treated. Scale bar = 100 **μ**m.

**Figure 5:**
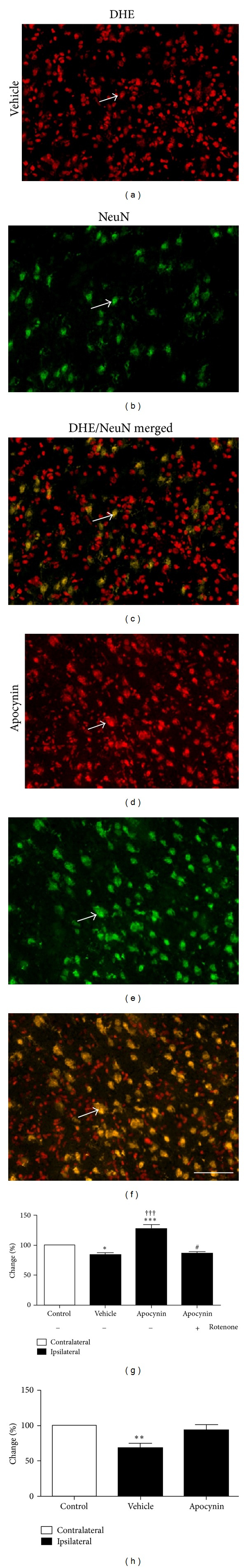
Immunohistochemical localization of neurons (NeuN) and concurrent superoxide generation (dihydroethidium) in stroke-affected brain following vehicle control ((a)–(c)) and apocynin ((d)–(f)) treatments. Fluorescence micrographs of the ipsilateral cortex with superoxide-sensitive DHE ((a), (d)), the neuronal antibody NeuN ((b), (e)), and merged images ((c), (f)). Arrows point to a typical neuron in the ischemic core. Quantification of neuronal specific superoxide release in the core infarct region detected *in situ* 3 days after stroke in the ipsilateral cortex (stroke affected; solid bars) compared with the contralateral cortex (nonaffected; open bar) following ischemic stroke in vehicle control; apocynin-treated rats; apocynin-treated rats coincubated in the presence of rotenone *in situ* (g). Quantification of neuronal specific superoxide release in the core infarct region in the ipsilateral striatum compared with the contralateral striatum in vehicle control and apocynin-treated rats (h). Relative fluorescence was quantified by tracing around cells double labelled with NeuN and analysed using ImageJ software. Data are presented as mean ± SEM (*n* = 11 apocynin treated; *n* = 9 vehicle treated). *P < 0.05, **P < 0.01, and ***P < 0.001 versus contralateral mirror image; ^†††^ *P* < 0.001 versus vehicle treated; ^#^ *P* < 0.01 versus apocynin-treated total ipsilateral fluorescence. Scale bar = 100 **μ**m.

To investigate the effect of cell-specific superoxide generation after stroke with apocynin treatment, sections were simultaneously labelled with dihydroethidium and the activated microglia/macrophage marker OX-42 (Figure 4), or dihydroethidium, and the neuronal marker NeuN (Figure 5). The majority of cells positive for dihydroethidium in the ipsilateral cortex in the vehicle-treated rats after stroke were colocalized with OX-42 (Figure 4(c)). Total dihydroethidium fluorescence from specific cells was again calculated by tracing around equal number of individual cells that were colabelled with each specific cell marker and the average calculated and compared to the average of the same number of similar cells within the contralateral mirror image.

Total dihydroethidium fluorescence from OX-42 double labelled cells within the ipsilateral cortex was significantly increased when compared with similar cells from the corresponding contralateral region (*P* < 0.01 ANOVA; Figure 4(g)). This increased dihydroethidium fluorescence from OX-42 positive cells in the ipsilateral cortex after stroke was reduced by approximately 51% in rats treated with apocynin (*P* < 0.001 ANOVA; Figure 4(g)). Similarly, treatment with apocynin also attenuated increased dihydroethidium fluorescence from OX-42 positive cells in the ipsilateral striatum compared with vehicle-treated controls (*P* < 0.01 ANOVA; Figure 4(h)).

The remaining cells positive for dihydroethidium in the core ipsilateral cortex of vehicle- and apocynin-treated rats after stroke were colocalized with NeuN (Figure 5(c)). Rats treated with apocynin appeared to have a greater number of surviving neurons in the ipsilateral cortex when compared with vehicle-treated rats (Figures 5(e) and 5(b)). To account for the disparity in the number of NeuN positive cells between the groups a constant number of randomly selected individual cells co-labelled with dihydroethidium was sampled (*n* = 15 cells per region). Dihydroethidium fluorescence from individual surviving NeuN positive cells from vehicle-treated rats was significantly reduced in the ipsilateral cortex when compared with similar cells from the corresponding contralateral region (*P* < 0.05, ANOVA, Figure 5(g)). In contrast, dihydroethidium fluorescence from individual neurons from apocynin-treated rats was significantly increased by approximately 27% in the ipsilateral cortex after stroke when compared with similar cells from the contralateral region (*P* < 0.001 AVOVA; Figure 5(f)). Coincubation of dihydroethidium with the mitochondrial inhibitor, rotenone, attenuated increased ROS detected in neurons from the ipsilateral cortex of apocynin-treated rats to levels equivalent to those of neurons from the ipsilateral cortex of vehicle-treated controls (*P* < 0.01 ANOVA; Figure 5(g)). 

There were few surviving neurons within the ipsilateral striatum in both treatment groups. Rats treated with vehicle and cells positive for NeuN had significantly less dihydroethidium fluorescence in comparison to the contralateral mirror region (*P* < 0.01 ANOVA; Figure 5(h)). In contrast, there was no difference in dihydroethidium fluorescence in NeuN positive cells from the ipsilateral striatum compared with the contralateral mirror region from apocynin-treated rats (Figure 5(h)).

### 3.6. Nox2 Immunoreactivity

**Figure 6:**
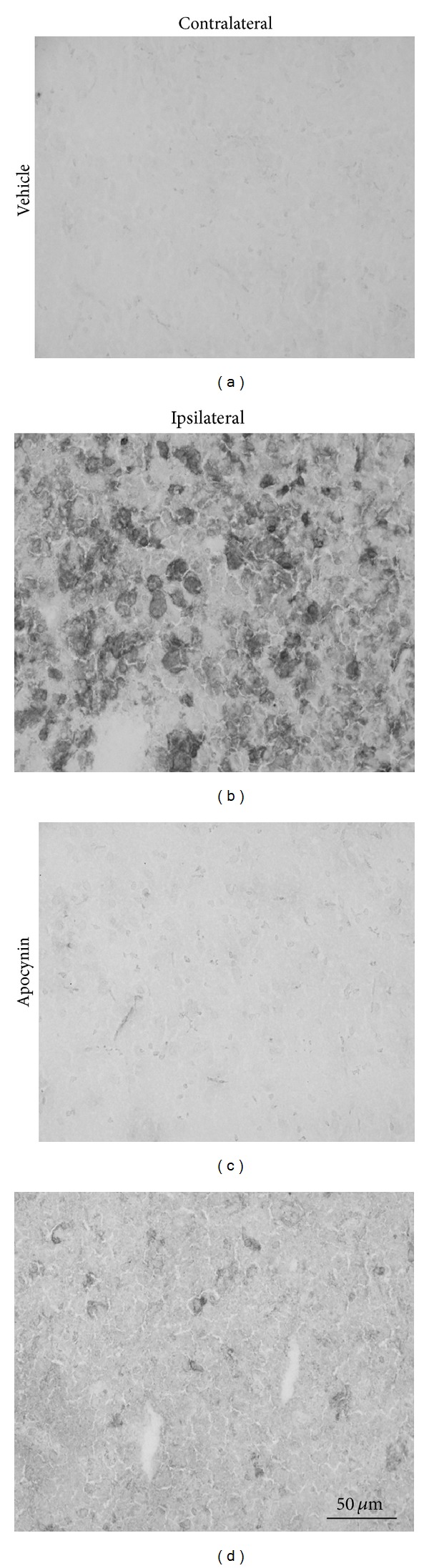
Immunohistochemical localization of cortical NADPH oxidase subunit Nox2 in the stroke-affected cortex (b), (d) and the contralateral mirror image ((a), (c)), in vehicle control ((a), (b)) and apocynin- ((c), (d)) treated rats. Micrographs taken from the core infarct region 3 days after stroke show a reduction in Nox2 positive cells following apocynin treatment (d) compared with vehicle control (b). Scale bar = 50 **μ**m.

To investigate further differential effects of apocynin treatment on individual cellular responses to ROS generation following stroke, changes in Nox2 protein expression were examined. A clear increase in Nox2 immunoreactivity was detected within the core infarct of the ipsilateral cortex (Figure 6(b)) in vehicle-treated rats after stroke when compared with the corresponding contralateral region (Figure 6(a)). This increase in immunoreactivity appeared to be concentrated within cell membranes of amoeboid-like cells within the ipsilateral cortex. In rats treated with apocynin, Nox2 immunoreactivity within the core infarct of the ipsilateral cortex was markedly reduced in comparison to vehicle-treated ones (Figure 6(d)).

Similar increases in Nox2 immunoreactivity were also detected in the core infarct of the ipsilateral striatum of vehicle treated rats following stroke when compared with the corresponding contralateral region, an effect that was again absent in apocynin-treated rats (data not shown).

### 3.7. OX-42 Stereology

**Figure 7:**
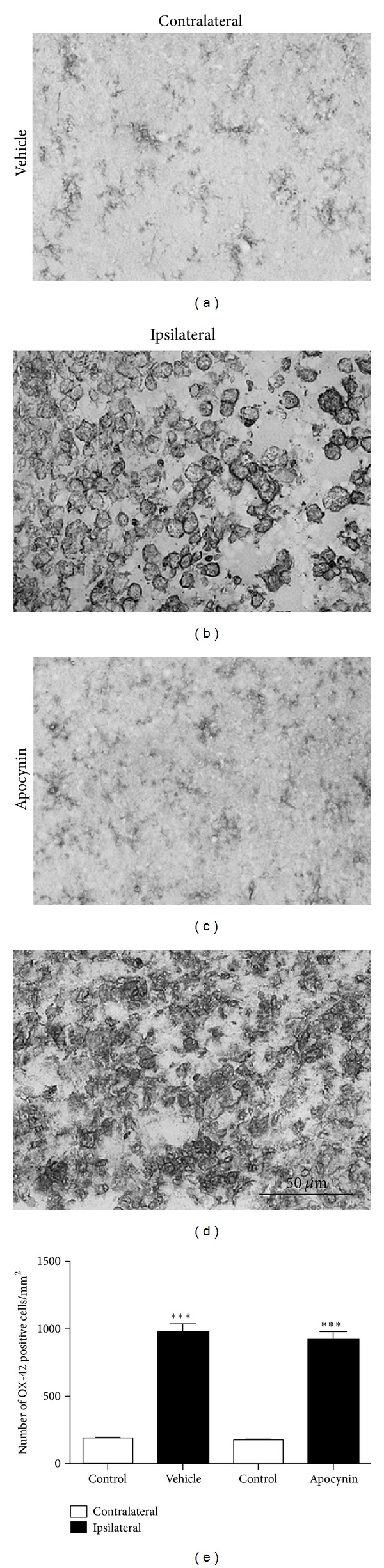
Immunohistochemical localization of activated microglia/macrophages by OX-42 in the stroke-affected cortex ((b), (d)) and the contralateral mirror image ((a), (c)), in vehicle control ((a), (b)) and apocynin- ((c), (d)) treated rats. Cell counts of micrographs taken from the stroke-affected cortex show no effect on the number of OX42 positive cells between vehicle control and apocynin treatment (e). ***P < 0.001 versus contralateral control. Scale bar = 50 **μ**m.

In order to determine if reduced Nox2 protein expression in apocynin-treated rats following stroke was associated with changes in inflammatory cell numbers, light field OX-42 immunohistochemistry was utilized to compare the number of activated microglia/macrophages in the infarct core of the ipsilateral cortex where an equivalent area of damage was demonstrated in both vehicle- (Figure 7(a)) and apocynin-treated rats (Figure 7(b)). Cell counts of OX-42 immunopositive cells after stroke showed an increase in the number of activated microglia/macrophages in the core infarct of the ipsilateral cortex from both vehicle- (*P* < 0.001 ANOVA; Figure 7(c)) and apocynin-treated rats when compared to their respective contralateral regions (*P* < 0.001 ANOVA; Figure 7(c)). There was no difference in the number of activated microglia/macrophages appearing within the infarcted ipsilateral cortex between treatment groups (Figure 7(c)).

## 4. Discussion

Increased levels of reactive oxygen species, in particular superoxide, are a major cause of cellular damage and lesion progression following cerebral ischemia. One of the most important sources of superoxide in mammals is NADPH oxidase [10]. In the present study, we report that treatment with a high dose of the antioxidant and Nox2 NADPH oxidase inhibitor, apocynin, reduces cortical damage after endothelin-1 stroke and reperfusion. We also confirm that apocynin treatment reduces ROS generation in activated microglia/macrophages, but importantly we report a novel finding that apocynin treatment reduces Nox2 expression after stroke without effect to inflammatory cell numbers. Additionally, we also report for the first time differential responses to cell-specific ROS generation *in vivo* following treatment with apocynin, in particular an unexpected increase in neuronal ROS 3 days after stroke, compared to vehicle controls. This study highlights the need to investigate the mechanisms through which individual cells respond to apocynin and treatments that may target specific NADPH oxidases.

Stereotaxic application of endothelin-1 to the middle cerebral artery is now a well established method of focal ischemia in conscious rats [22] with recent adaption of the model for use in primates [23]. This is the first study to examine the effects of apocynin in an animal model where stroke is induced in the conscious rat, where treatment afforded protection to the cortex 3 days after reperfusion, but failed to reduce damage to subcortical structures. In contrast, studies using anesthetized animals to induce stroke report significant protection to the striatum in both rats [12] and mice [13, 14] following treatment with apocynin. The use of anesthetics can confound experimental findings due to their own cytoprotective effects [24–26], which include reduction in reactive oxygen species. The endothelin-1 model is the only model of stroke in animals that avoids the use of general anesthetics and their neuroprotective side effects, which may account for the lack of effect observed in the striatum with apocynin.

Another consideration for lack of effect in the striatum is the time in which infarct assessment has been conducted. Previous reports assessing the cytoprotective effects of apocynin measured infarcts only up to 24 hours after reperfusion, at times when the infarct may not have fully matured in the models used for assessment [12–14, 27]. Indeed we have shown previously that ischemic/reperfusion injury does not reach maximal until 3 days after stroke in rats with middle cerebral artery occlusion [28]. Hence, the lack of protection observed in the striatum observed in the present study may simply be associated with the progression of injury over a longer time period. This highlights an important need to examine the effects of all potential neuroprotective agents such as apocynin, over extended recovery times to ensure that apocynin does not merely delay ischemic damage, rather than prevent it. 

NADPH oxidase and inflammation following stroke has been studied in more detail in recent years for its contribution to the expansion of the core lesion in the days following reperfusion [29]. In the present study, treatment with apocynin reduced superoxide generation within inflammatory cells up to the 3 days after stroke, but most importantly, we show for the first time that apocynin treatment also attenuated increased Nox2 associated with activated microglia/macrophages, without change in the number of cells. This supports previous findings *in vitro* where apocynin attenuated superoxide release from neutrophils and macrophages without effect to inflammatory cell activation [30, 31]. Collectively, these results suggest that apocynin reduces the inflammatory response, not through reduction of inflammatory cell activation, but potentially through specific effects on Nox2 expression and resulting ROS generation from these cells.

The mechanism of action through which apocynin attenuates ROS has been the topic of current debate with a report suggesting that apocynin is an antioxidant rather than a specific inhibitor of NADPH oxidase, at least in the vasculature [32]. Others have demonstrated that apocynin is a direct inhibitor of the Nox2 NADPH oxidase through prevention of NADPH oxidase subunit assembly [33, 34]. If indeed apocynin does inhibit the Nox2 subunit assembly, and given our results which show reduced Nox2 expression after stroke and treatment with apocynin, it would be interesting to explore the role of Nox2 subunit assembly in the upregulation of Nox2 during inflammatory events. It is also unlikely that apocynin in the present study is acting as an antioxidant given that we show an unexpected increase in neuronal superoxide in apocynin-treated rats, an effect that was not seen with vehicle treatment, and would suggest a lack of antioxidant activity. Our data are also in direct contrast to previous studies using the free radical scavenger, Edaravone, where increased dihydroethidium detected ROS from NeuN positive cells was attenuated with antioxidant treatment following permanent stroke in mice [19]. Since apocynin treatment has not reduced neuronal superoxide generation in the present study, this would suggest that it is not acting as purely as an antioxidant. Moreover, a recent study where apocynin reduced superoxide generation and subsequent damage following stroke also showed that apocynin treatment afforded no additional protection in Nox2 −/− mice [14], again supporting a specific effect on Nox2 generated ROS alone by apocynin within the brain after stroke.

The differential response to superoxide in the current study highlights the importance of investigating individual cell responses to superoxide *in vivo* following stroke. Differential effects on superoxide generation have been observed with apocynin *in vitro*: Vejražka and colleagues [35] also report opposing effects of apocynin with phagocytes and nonphagocytic cells, where apocynin acted as a superoxide promoter in vascular cells. The mechanism through which apocynin treatment results in increased superoxide generation in neurons 3 days after stroke in the present study is yet to be determined. However, when incubated with macrophages *in vitro*, apocynin initially caused an increase in ROS production that was quickly followed by ROS inhibition [35]. This effect was accounted for by the presence of myeloperoxidase (MPO) in phagocytes that is reported to convert apocynin into active dimers that are then responsible for Nox inhibition. Endothelial cells and smooth muscle cells do not contain MPO perhaps accounting for sustained increases in ROS generation in apocynin-treated cells [35]. In the healthy brain, neurons do not normally express MPO, although neuronal expression of MPO has been detected in Alzheimer's disease [36]. Despite this, no MPO expression has been demonstrated in neurons during ischemia and reperfusion, and further studies could be undertaken to determine if the opposing actions of apocynin in neurons is due to the absence of MPO.

A more likely explanation for increased neuronal superoxide in apocynin-treated rats could be related to the progression of injury over time. We previously reported increases in superoxide generation within activated neurons from the core infarct 6 hours after stroke, an effect that was later observed in the outer penumbra by 24 hrs, and absent by 3 days once the infarct had matured and neurons lost [4]. This increased neuronal superoxide was partly attenuated by the mitochondrial inhibitor rotenone, but was unchanged by catalase, L-NAME or allopurinal, [4]. In the present study, we observed a similar effect where increased superoxide release in the ipsilateral cortex was detected in neurons from apocynin treated rats, an effect that was attenuated in the presence of rotenone to levels similar to that of neurons in the ipsilateral cortex from vehicle controls. Hence, stroke-induced increase in ROS release from neurons of apocynin treated rats appears to be associated with changes in mitochondrial respiration and not due to compensatory changes in other neuronal NADPH oxidases, such as Nox4, which has recently been reported as the major contributor to neuronal oxidative stress following stroke in mice [37]. The detection of mitochondrial-induced oxidative stress within neurons from apocynin-treated rats after stroke further supports our hypothesis of an expanding infarct that has been delayed due to Nox2 inhibition in inflammatory cells. It would also explain the lack of increase in neuronal ROS detected within the ipsilateral striatum of apocynin-treated rats where damage appears to have reached maximum.

Ours is the first study to examine the effects of apocynin beyond a 24 hr recovery period after stroke and reperfusion. Given that changes in neuronal oxidative stress 3 days after stroke in apocynin-treated rats appear not to be the result of increased NADPH oxidase activity, it is likely that inhibition of Nox2 does not prevent the expansion of the lesion but merely delays its progression. Studies have now demonstrated that treatments targeting oxidative stress and inflammation should be assessed over longer time periods because although a compound may be initially effective with a short survival time, beneficial effects may be lost with longer periods of recovery [38]. Indeed this may account for why so many promising results in animal models have failed to progress to clinical success since patients often survive a stroke episode and recovery is monitored for years after treatment.

## 5. Conclusion

Using a rat model of conscious stroke, we report for the first time that apocynin treatment, prior to and after stroke, reduces inflammatory cell-mediated superoxide generation through attenuation of increased Nox2 expression that occurs in response to stroke-induced brain injury. Additionally, we show that neurons in the cortex of apocynin-treated rats are in a state of oxidative stress indicative of progression of injury suggesting that targeting Nox2 after stroke merely delays the progression of brain injury rather than prevent it. The outcomes of this study in comparison to previous work highlight the importance of preclinical testing of potential cytoprotective agents in multiple species, in more than one model of stroke, but most importantly with inclusion of extended recovery times that mimic more closely the human scenario.
